# The Impact of Carrot Enriched in Iodine through Soil Fertilization on Iodine Concentration and Selected Biochemical Parameters in Wistar Rats

**DOI:** 10.1371/journal.pone.0152680

**Published:** 2016-04-04

**Authors:** Ewa Piątkowska, Aneta Kopeć, Renata Bieżanowska-Kopeć, Mirosław Pysz, Joanna Kapusta-Duch, Aneta Agnieszka Koronowicz, Sylwester Smoleń, Łukasz Skoczylas, Iwona Ledwożyw-Smoleń, Roksana Rakoczy, Edyta Maślak

**Affiliations:** 1 Department of Human Nutrition, Faculty of Food Technology, University of Agriculture in Krakow, Balicka,122, 30–149, Krakow, Poland; 2 Unit of Plant Nutrition, Institute of Plant Biology and Biotechnology, Faculty of Biotechnology and Horticulture, University of Agriculture in Krakow, al. 29 Listopada 54, 31–425, Krakow, Poland; 3 Department of Fruit, Vegetable and Mushroom Processing, Faculty of Food Technology, University of Agriculture in Krakow, Balicka 122, 30–149, Krakow, Poland; 4 Unit of Biochemistry, Institute of Plant Biology and Biotechnology, Faculty of Biotechnology and Horticulture, University of Agriculture in Krakow, Al. 29 Listopada 54, 31–425, Krakow, Poland; 5 Jagiellonian Centre for Experimental Therapeutics (JCET), Jagiellonian University, Bobrzynskiego 14, 30–060, Krakow, Poland; Oklahoma State University, UNITED STATES

## Abstract

Iodine is one of the trace elements which are essential for mammalian life. The major objective of iodine biofortification of plants is to obtain food rich in this trace element, which may increase its consumption by various populations. Additionally, it may reduce the risk of iodine deficiency diseases. In this research for the first time we have assessed the bioavailability of iodine from raw or cooked carrot biofortified with this trace element on iodine concentration in selected tissues and various biochemical parameters as well as mRNA expression of some genes involved in iodine metabolism in Wistar rats. Statistically, a significantly higher iodine level was determined in urine, faeces and selected tissues of rats fed a diet containing biofortified raw carrot as compared to a diet without iodine and a diet containing control cooked carrot. Biofortified raw carrot significantly increased triiodothyronine concentration as compared to animals from other experimental groups. The highest thyroid stimulating hormone level was determined in rats fed control cooked carrots. mRNA expression of selected genes was affected by different dietary treatment in rats’ hearts. Biofortified raw and cooked carrot could be taken into account as a potential source of iodine in daily diets to prevent iodine deficiency in various populations.

## Introduction

In recent years, the world population has not always been suffering due to low calorie intake, but rather due to inadequate intake of selected nutrients in its daily diet, especially trace elements, including iodine and iron. Additionally, malnourished people often eat meals which are based on staple crops and, consequently, have little access to another kind of food, e.g. a wide range of food of animal as well as plant origin—necessary for a proper nutrition [1].

Iodine is an essential trace element which is necessary for production the thyroid gland hormones (3,5,3’,5’-tetraiodo-L-thyronine, T_4_; 3,5,3’-triiodo-L-thyronine, T_3_). They are crucial for mammalian life [2]. It is present in human body in minute amounts (15–20 mg) (almost exclusively in thyroid gland). The bioavailability of iodide depends on oral intake, and the recommended daily intake is dependent on the age and physiological condition of humans. However, iodine is also absorbed from air, though mucous membranes of respiratory system, and though skin [3]. The recommended daily allowance for pre-school children is 90 μg, 120 μg for school children, 150 μg for adolescents and adults, and 250 μg for pregnant and lactating women respectively [4].

Natural iodine content of most foods and beverages is low. The most commonly consumed foods provide 3 to 80 μg in daily diet [5, 6]. The major dietary sources of iodine in the USA, Europe and Australia are bread, milk and to a lesser extent seafood [7, 8].

In nearly all countries where iodine deficiency occurs, it is now well recognized that universal salt iodization (USI) is the most effective way to achieve the elimination of iodine deficiency diseases (IDD). Although USI has successfully reduced IDD in many countries, albeit more in developed than in developing countries, a third of the global population is still unprotected from iodine deficiency [9]. Low dietary iodine intake may lead to goiter and many other IDD (e.g. infant mortality, endemic cretinism, impaired mental function, delayed physical development) [10–12].

It seems that biofortification of staple foods, for example commonly consumed vegetables, is a proper strategy to eliminate iodine deficiency [13, 14]. The major objective of iodine biofortification of plants is to obtain food rich in this trace element, which may increase its consumption by various populations. Additionally, it may reduce the risk of IDD. Our previous study has shown that biofortified lettuce can be considered as a good source of bioavailable iodine [15]. Carrot is a very popular root vegetable in many countries both in Europe and in North America. This vegetable can be consumed raw or cooked; what is more, it can be a potential source of various nutrients. Iodine biofortification of a carrot during growth may be a good source of this trace element [16].

The objective of this study was to assess the effect of adding of raw or cooked carrot biofortified with iodine, in potassium iodide form, to the experimental diets of Wistar rats. The following parameters were considered: iodine content in selected tissues, lipid profile, thyroid hormone concentration and mRNA expression of selected genes involved in iodine metabolism.

## Materials and Methods

### Plant material

Carrot ‘Kazan F_1_‘ cv. was cultivated in 2013 in a field experiment on heavy soil (2% sand, 48% dust and 50% loam) characterized by: pH(H_2_O) 7.77, pH(KCl) 7.35, EC (electrical conductivity) 0.12 mS·cm^-1^ and 3.14% content of organic matter -and following content of macroelements: 0.2mg N-NH_4_ (nitrogen-ammonium), 6.4 mg N-NO_3_ (nitrogen-nitrate), 1.1 mg P (phosphorous), 17.6 mg K (potassium), 97.6 mg Mg (magnesium), 6 668.5 mg Ca (calcium) and 137.1 mg S (sulphur) in 1 dm^3^ of soil. A field study with carrot (*Daucus carrota* L.) cv. 'Kazan F_1_' cultivation was conducted in Marszowice (50°18’6 N, 20°09’1 E), near Krakow, Poland. Based on soil analysis, before sowing, mineral fertilizers such as: urea, potassium chloride and potassium monophosphate were introduced into soil in order to supplement the deficiency of nutrients to the optimal level for carrot (in mg·dm^-3^ of soil): N-100, P-80 and K-200. Pre-sowing fertilization with Mg, Ca and S was not performed, because their content in soil covered the nutritional requirements of carrot.

This part of the study included: 1) control carrot–grown without iodine fertilization and 2) carrot grown on soil fertilized with KI (potassium iodide). Potassium iodide was applied twice: before cultivation and as a top-dressing at canopy closure (each as 2.5 kg I·ha^-1^) in a total dose of 5 kg I·ha^-1^. Carrots were cultivated in one row on 40 cm wide and 30 cm high ridges at a seeding rate of 37 seeds m^-1^ (approximately 600 000 seeds per hectare). The seeds were sown on 24^th^ of April 2013. The experiment was arranged in a split-plot design with four replications of 6 m × 4 m (24 m^2^) plots. For the animal study, randomly selected full grown carrot roots were collected at harvest (23^th^ of September 2013) from the middle part of each plot, individually for both treatments.

### Cooking Method

Fresh, not peeled carrots (biofortified and not biofortified with iodine) were cleaned, washed and then cooked in a laboratory in distilled deionized (dd) water. Vegetables were put into boiling dd water (without salt) in a covered stainless steel pot (1:5, carrot/water) and cooked on a moderate flame. Cooking time was measured when, after putting the vegetables in, the water started boiling again. Cooking time was 20 min. Samples were then cooled down and frozen in -20°C and stored until the freeze drying process.

### Analysis in plant material

Fresh samples of carrot were frozen and freeze-dried with lyophilizer (Christ Alpha 1–4, Gefriertrocknungsanlangen, Germany). In the freeze-dried samples the concentration of proteins, raw fat, total dietary fiber and ash was measured according to AOAC [17] methods. Carbohydrates were calculated as previously reported [15].

In order to analyze the iodine content, air-dried carrot root samples were ground in a variable speed rotor mill Pulverisette 14 FRITSCH (Idar-Oberstein, Alemania, Germany) using a 0.5 mm sieve. Digestion of 0.5 g samples of carrot in the mixture of 10 cm^3^ 65% nitric acid (HNO)_3_ (superpure, Merck, Whitehouse Station, NJ, USA) and 0.8 cm^3^ 70% perchloric acid (HClO_4_) (superpure, Polskie Odczynniki Chemiczne, Gliwice, Poland) was conducted in the microwave system CEM MARS-5 Xpress (CEM World Headquarters, Matthews, NC, USA). The content of iodine was analyzed through the cold vapour generation technique with use of high-dispersion Inductively Coupled Plasma Optical Emission Spectrometry (ICP-OES; Prodigy spectrometer–Leeman Labs New Hampshire, MA, USA) [18, 19]. A similar method was used for the determination of iodine in the experimental diets for rats.

### Animal study

Five week old male Wistar rats (n = 48), with average body mass 129±10 g were purchased from the Animal Husbandry in Brwinów, Warsaw, Poland. Experimental procedures were approved by the First Local Ethical Committee on Animal Testing at the Jagiellonian University in Krakow (Poland, res. no 103/2012). Before the experiment, the rodents were acclimatized for one week with a standard laboratory chow. After the acclimatization period the rodents were randomly divided into six experimental groups (n = 8). Experimental diets were prepared based on AIN-93G diets [20]. A detailed description of diets is reported in Table 1. In the C-diet (control diet), the mineral mixture contained iodine in the amount recommended by Reeves [20]. The diet without iodine (DWI) was prepared with a mineral mixture without iodine. In the diet containing biofortified raw carrot (BFRC- diet with biofortified raw carrot) the only source of iodine was carrot (mineral mixture did not contain iodine). In the diets 4–6 the mineral mixture did not contain iodine and the sources of iodine were carrots (Table 1). Group 4 was fed a diet with control raw carrot (RCC), group 5 received a diet with biofortified cooked carrot (BFCC) and group 6 with control cooked carrot (CCC). The rodents were housed separately in stainless steel metabolic cages at 21°C and 12/12 h–light/dark cycle. During the experiment, animals had free access to deionized distilled water. The intake of experimental diets were recorded every day. Body weight gain was recorded during the whole experiment on weekly basis. Urine and faeces were collected between the 7^th^-11^th^ and 22^nd^-27^th^ days of the experiment (II^nd^ and IV^th^ week of experiment, respectively) to assess iodine excretion. Collected samples were stored at -20°C until the analysis time.

**Table 1:** Experimental diets composition.

Ingredient	C-diet	DWI	BFRC	RCC	BFCC	CCC
Corn starch	532.486	532.486	521.116	444.836	516.656	517.026
Saccharose	100	100	100	100	100	100
Casein	200	200	200	200	200	200
Soybean oil	70	70	70	70	70	70
Fiber	50	50	45.17†	10.45†	43.73†	43.36†
Mineral mix^1^	35	35*	35*	35*	35*	35*
Vitamin mix^1^	10	10	10	10	10	10
choline chloride	2.5	2.5	2.5	2.5	2.5	2.5
TBHQ**	0.014	0.014	0.014	0.014	0.014	0.014
Raw biofortified with KI carrot	0	0	16.2			
Raw control carrot	0	0		127.2		
Cooked biofortified with KI carrot	0	0			22.1	
Cooked control carrot	0	0				22.1

*mineral mix without iodine; in these diets the source of iodine was biofortified carrot or control carrot

**tert-butylhydroquinone

^1^ according to AIN-93G

† fiber was delivered from control or biofortified carrots

C diet—control AIN-93G diet; DWI—diet without iodine in mineral mix; BFRC—diet containing biofortified raw carrot; RCC—diet containing control raw carrot; BFCC—diet containing biofortified cooked carrot; CCC—diet containing control cooked carrot

After 4 weeks of experimental period fasted rats were anaesthetized (substance used—izofluran 4%; inhaled). Blood was obtained by heart puncture and collected in plain test tubes. Blood samples were collected to obtain serum by centrifugation (1500 x *g*, 15 min.). Livers, kidneys, thyroid glands and hearts were dissected, washed in 0.9% sodium chloride, dried with laboratory tissue paper and weighed. Serum and tissue samples were kept frozen at -80°C until the analysis.

### Analysis in serum and blood

The serum was analyzed in order to measure the concentration of total cholesterol—TC; (cat no. Liquick Cor-CHOL60 2–204, PZ Cormay S.A. Lublin, Poland), HDL-cholesterol (cat no. Cormay HDL 2–052, PZ Cormay S.A. Lublin, Poland), and triacylglycerols—TAG (cat no. Liquick Cor-TG60 2–253, PZ Cormay S.A. Lublin, Poland). The differences between TC and HDL were used for calculations of the LDL+VLDL level [21]. The concentration of thiobarbituric acid reactive substances (TBARS) was measured with the OxiTekTBARS kit (cat no. 850-287-KI01, Zeptometrix, Bufallo, NY, USA). The level of triiodothyronine (T3) and thyroxine (T4) was measured with the Mouse/Rat kits (cat no. T3043T-100; T4044T-100; respectively, Calbiotech, Spring Valley, CA, USA). The level of the thyroid stimulating hormone (TSH) was measured with the Rat kit (cat no. CEA463Ra, Cloud-Clone Corp., Houston, TX, USA). The level of glucose was measured in the whole blood with a glucometer (Accu-chek, Roche Diagnostic, Mannheim, Germany). The activity of the aspartate aminotransferase (AST) and the alanine aminotransferase (ALT) in the serum was measured using the Alpha Diagnostic kits (Alpha Diagnostic, Warsaw, Poland; cat no. A6661-050, A6624-050, respectively).

### Iodine content in urine, faeces and selected tissues

Collected samples of urine were adjusted to the same volume before analysis. The faeces, kidneys, livers, hearts and femoral muscles were freeze-dried. After freeze-drying, the organs were weighed and crushed in a mortal and pestle. Then the prepared samples (particle size about 1 mm) were used for measurements of iodine content. The content of iodine in these samples was analyzed by the cold vapor generation technique with the use of ICP-OES Prodigy spectrometer (Leeman Labs, New Hampshire, MA, USA) ([17, 18] after sample digestion in the mixture of 10 cm^3^ 65% HNO_3_ (superpure, Merck, Whitehouse Station, NJ, USA) and 0.8 cm^3^ 70% HClO_4_ (superpure, Polskie Odczynniki Chemiczne, Gliwice, Poland) in the microwave system CEM MARS-5 Xpress (CEM World Headquarters, Matthews, NC, USA).

### The Gene expression

RNA was isolated from the thyroid glands, livers, kidneys and hearts with a commercially available kit (cat no. 036–100, Total RNA Mini Plus A&A Biotechnology, Gdynia, Poland). RNA content was determined by a spectrophotometer (Multiscan Go, Thermoscientific, Waltham, MA USA) using absorbances at 260 and 280 nm. For cDNA synthesis, RNA was reversely transcribed with the use of the TranScriba cDNA Synthesis Kit, (cat no. 4000–100 A&A Biotechnology, Gdynia, Poland). cDNA was subjected to real-time PCR (CFX96 Touch™ Deep Well Real-Time PCR Detection System Bio Rad, Hercules, CA, USA) in a reaction of a mixture containing the TaqMan Gene Expression Master mix (cat no.4369016, Applied Biosystems, Foster City, CA, USA) and primers for the following genes: deiodinase iodothyronine type 1 (Dio1), E2F transcription factor 1 (E2f1), thyroid hormone receptor alpha (Thra) and thyroid hormone receptor beta (Thrb) with fluorescent marked starters (Invitrogen, Life Technologies, Oslo, Norway) as it was previously described [14]. Expression rates were calculated as normalized quantification cycle (Cq) difference between the control and the sample with an adjustment for amplification efficiency relative to the expression level of the reference gene *18S*.

### Histological analysis

Thyroid glands were fixed in 4% buffered formalin and prepared according to the standard paraffin method. Five μm sections were stained with hematoxylin and eosin (H&E) for general histology and with periodic acid–Schiff (PAS) for glycoprotein visualization. Sections were photographed under the 400x magnification by Olympus BX51 light microscope equipped with the VS-120 virtual slide scanning system and VC50 camera (Olympus, Germany). The size of follicles, the height of the epithelial cells as well as the amount and staining quality of the colloid were analyzed to measure the secretory activity of the thyroid gland.

### Statistical analysis

The data were presented as mean ± SD. One-way, the analysis of variance was used for testing the difference at P≤0.05 (Statistica v. 10.0, StatSoft, Inc., Tulsa, OK, USA). The Duncan test was used for testing the differences between the experimental treatments.

## Results

### Basic chemical composition and iodine content in plant material

The highest level of protein was measured in control cooked carrots as compared to other carrots (Table 2). The highest concentration of digestible carbohydrates was analyzed in the cooked carrot biofortified with KI as compared to other experimental carrots.

**Table 2:** Basic chemical composition of carrot used for preparation of experimental diets (g/100 g d.m.).

	Control raw carrot	Raw biofortified with KI carrot	Control cooked carrot	Cooked biofortified with KI carrot
Protein	5.80±0.01^a^	8.40±0.09^b^	10.31±0.74^c^	5.67±0.21^a^
Crude fat	0.84±0.1^a^	1.08±0.01^b^	1.68±0.02^c^	1.81±0.07^d^
Digestible carbohydrates	57.35±0.01^c^	55.30±0.70^b^	51.52±0.79^a^	58.93±0.75^d^
Dietary fiber	31.10±0.26^a^	29.82±1.02^ab^	30.06±0.21^a^	28.37±0.21^b^
Ash	4.90±0.17^a^	6.42±0.14^b^	5.40±0.22^a^	5.22±0.26^a^
Iodine [mg/1000 g d.m.]	1.63±0.12^b^	12.81±0.44^d^	0.53±0.07^a^	9.37±0.28^c^

Values in rows with different letters (e.g. a, b, c) are significantly different, P≤0.05

The highest concentration of dietary fiber was found in control raw carrot compared to the cooked carrot biofortified with KI.

The highest concentration of ash was measured in the raw carrot biofortified with KI compared to other experimental groups. The highest level of iodine was measured in control raw carrot as well as in cooked carrot biofortified with KI.

### Body weight gain, weight of selected organs

The highest body weight gain was measured in rats fed diets containing control raw carrots (RCC diet) and cooked carrots biofortified with iodine (BFCC diet) as compared to other experimental groups. The highest fed efficiency ratio (FER) was found in rats fed raw control carrots and cooked carrots biofortified with iodine in comparison to rodents fed control diet (C-diet) and AIN-93G diet without iodine (DWI diet) (Table 3). The lowest liver weight was measured in animals fed DWI diet as compared to rodents fed diet with raw biofortified carrot (BFRC diet), RCC as well as rats fed BFCC diet. The kidney weight was not affected by various dietary treatments. The highest heart weight was found in groups of rats fed C-diet as compared to other experimental groups with exception of rodents fed DWI diet. The highest weight of thyroid gland was measured in animals fed C-diet compared to other experimental groups.

**Table 3:** Body gain, fed efficiency ratio (FER) and weight of selected organs.

Treatment	C-diet	DWI	BFRC	RCC	BFCC	CCC
Body gain [g]	144.12±9.6^a^	144.87±7.0^a^	150.62±9.36^a^	158.78±15.47^b^	156.87±12.53^b^	147.37±10.11^a^
FER*	0.349±0.02^a^	0.352±0.02^a^	0.365±0.02^ab^	0.385±0.04^b^	0.380±0.03^b^	0.357±0.02^ab^
Liver [g]	10.04±1.11^ab^	9.61±0.71^b^	10.91±0.80^a^	10.81±1.25^a^	10.84±0.29^a^	10.26±1.09^ab^
Kidney ** [g]	2.31±0.30^a^	2.41±0.17^a^	2.36±0.08^a^	2.44±0.27^a^	2.43±0.18^a^	2.29±0.35a
Heart [g]	1.31±0.15^c^	1.20±0.11^bc^	1.08±0.06^a^	1.17±0.07^ab^	1.13±0.08^ab^	1.11±0.16^ab^
Thyroid gland [g]	0.23±0.08^b^	0.18±0.04^a^	0.16±0.02^a^	0.18±0.02^a^	0.18±0.04^a^	0.18±0.02^a^

Values in rows with different letters (e.g. a, b, c) are significantly different, P≤0.05

* FER feed efficiency ratio (g) (body weight gain/diet consumed (g)

**weight of both kidneys

C diet—control AIN-93G diet; DWI—diet without iodine in mineral mix; BFRC—diet containing biofortified raw carrot; RCC—diet containing control raw carrot; BFCC—diet containing biofortified cooked carrot; CCC—diet containing control cooked carrot

### Iodine excretion in urine, faeces and selected organs

The highest iodine excretion with urine in week II^nd^ and IV^th^ was measured in the group fed C-diet as compared to other experimental groups (Table 4). Additionally, in both weeks, the lowest level of iodine was measured in urine of rats fed diet without iodine (DWI), cooked carrots biofortified with iodine (BFCC) and control cooked carrots (CCC). The highest iodine excretion in week II was measured in faeces of rats fed C-diet as compared to other experimental groups. In week IV the highest iodine excretion was measured in faeces of rats fed C-diet and BFRC diet as well as BFCC diet as compared to the concentration of iodine in faeces of rodents fed DWI-diet and CCC diet (Table 4). The highest concentration of iodine in femoral muscle and kidney was measured in rodents fed C-diet and BFRC diet as compared to other experimental rats. Similar results were found in livers of experimental rats. Rats fed the BFRC diet had higher concentration of iodine in their hearts as compared to other experimental groups with the exception of hearts of rats fed C-diet.

**Table 4:** Concentration of iodine in urine, faeces, muscle and selected organs.

Treatment	C-diet	DWI	BFRC	RCC	BFCC	CCC
Urine μg/dm^3^						
week II	180.10±20.46^d^	45.48±5.85^a^	139.91±14.51^b^	128.93±4.27^b^	81.17±5.87^c^	48.35±6.44^a^
week IV	201.44±6.82^e^	53.45±9.81^a^	140.97±14.51^d^	117.47±4.56^c^	63.11±5.26^b^	46.47±3.32^a^
Faeces mg/kg d.m.						
week II	785.97±90.90^d^	117.00±6.89^a^	697.57±120.98^c^	554.51±98.26^b^	618.14±136.76^bc^	99.19±20.75^a^
week IV	970.84±270.00^b^	147.45±38.42^c^	858.71±158.62^ab^	728.20±76.82^a^	829.49±77.33^ab^	132.51±4.45^c^
Selected organs mg/kg d.m						
Femoral muscle*	1.99±0.70^c^	0.46±0.12^a^	2.19±0.20^c^	0.56±0.12^ab^	0.91±0.28^b^	0.58±0.18^ab^
kidney*	8.64±0.96^c^	4.12±0.58^a^	9.22±1.12^c^	6.64±0.76^b^	6.85±0.79^b^	4.12±0.38^a^
liver*	4.46±0.83^c^	1.15±0.28^a^	4.37±1.14^c^	3.51±1.10^b^	2.94±0.43^b^	1.37±0.27^a^
heart*	3.57±0.28^bc^	1.43±0.27^d^	3.88±0.53^c^	0.61±0.22^a^	3.32±0.45^b^	0.93±0.24^a^

Values in rows with different letters (e.g. a, b, c) are significantly different, P≤0.05

*μg/kg d.m.

C diet—control AIN-93G diet; DWI—diet without iodine in mineral mix; BFRC—diet containing biofortified raw carrot; RCC—diet containing control raw carrot; BFCC—diet containing biofortified cooked carrot; CCC—diet containing control cooked carrot

### Selected biochemical parameters

The level of TC was not affected by various dietary treatments (Table 5). Rats fed the diet containing raw control carrots (RCC diet) as well as rodents fed CCC diet had significantly higher concentration of HDL in the serum as compared to the other experimental groups. It was also discovered that animals fed BFRC fed animals had a significantly higher concentration of HDL in the serum as compared to rats fed C- or DWI diets. The lowest concentration of LDL+VLDL cholesterol was measured in the serum of rats fed RCC and CCC diets as compared to the serum of rodents fed DWI diet. The highest concentration of TAG was measured in the serum of rats fed BFRC diet as compared to rodents fed C- and DWI diets (Table 5). The highest concentration of TBARS was measured in the serum of rats fed BFCC diet as compared to other experimental groups. The lowest concentration of T3 and T4 was measured in the serum of rats fed CCC diet as compared to other experimental groups. The highest concentration of TSH was measured in the serum of rats fed CCC diet as compared to rats fed C-diet as well as DWI diet. The level of glucose in blood was not affected by various dietary treatments. The highest concentration of the ALT was measured in the serum of rats fed BFCC or CCC diets.

**Table 5:** Selected biochemical parameters in serum of experimental rats.

Treatment	C-diet	DWI	BFRC	RCC	BFCC	CCC
TC [mmol/L]	1.65±0.16^a^	1.63±0.29^a^	1.76±0.26^a^	1.76±0.17^a^	1.77±0.36^a^	1.70±0.35^a^
HDL [mmol/L]	0.91±0.11^b^	0.98±0.13^bc^	1.16±0.11^cd^	1.39±0.14^a^	1.27±0.32^ad^	1.37±0.17^a^
LDL+VLDL [mmol/L]	0.74±0.16^a^	0.65±0.36^b^	0.60±0.31^ab^	0.36±0.19^a^	0.50±0.24^ab^	0.33±0.17^a^
TAG [mmol/L]	0.46±0.07^a^	0.46±015^a^	0.68±0.13^b^	0.63±0.22^ab^	0.50±0.08^ab^	0.58±0.28^ab^
TBARS [nmol/mL]	4.44±2.13^b^	7.80±3.14^ab^	9.61±3.76^a^	9.84±1.46^a^	15.66±2.16^c^	10.98±4.15^a^
T3 [μg/dL]	1.72±0.52^a^	1.87±0.48^a^	2.32±0.66^d^	1.50±0.54^a^	1.25±0.24^b^	0.68±0.40^c^
T4 [ng/mL]	10.18±1.83^a^	10.48±2.64^a^	11.22±1.46^a^	10.74±2.19^a^	9.68±2.05^a^	7.77±1.51^b^
TSH [ng/mL]	4.31±0.12^a^	4.27±0.07^a^	4.34±0.14^ab^	4.38±0.11^ab^	4.37±0.13^ab^	4.41±0.11^b^
Glucose [mg/dL]*	123±6.88^a^	122±4.34^a^	125±4.88^a^	125±10.73^a^	127±12.23^a^	123±4.00^a^
ALT [U/L]	9.38±3.55^a^	8.07±1.66^a^	11.71±3.37^a^	10.83±3.65^a^	24.37±5.07^b^	27.35±12.52^b^
AST [U/L]	45.68±7.55^a^	23.57±6.38^b^	67.73±27.33^a^	62.27±19.59^a^	56.45±10.77^a^	67.44±16.17^a^

Values in rows with different letters (e.g. a, b, c) are significantly different, P≤0.05

*In whole blood

C diet—control AIN-93G diet; DWI—diet without iodine in mineral mix; BFRC—diet containing biofortified raw carrot; RCC—diet containing control raw carrot; BFCC—diet containing biofortified cooked carrot; CCC—diet containing control cooked carrot

The lowest level of AST was found in the serum of rodents fed DWI diet as compared to other experimental groups.

### mRNA gene expression

Changes in mRNA expression of Dio1, E2f1, Thrα, and Thrβ were not detected in kidney and thyroid gland (data not shown). mRNA expression of Dio1, E2f1, Thrα, and Thrβ in heart was affected by different dietary treatments. The significantly highest Dio1 mRNA expression occurred in BFCC group. E2f1 mRNA expression significantly decreased in groups with carrot addition as compared to C and DWI groups. Thrα, and Thrβ mRNA expression was significantly highest in C group. In liver only some changes in Dio1 mRNA expression were detected. The highest level of mRNA expression was in BFCC and CCC groups as compared to C group (Table 6).

**Table 6:** Selected relative gene expression in liver and in thyroid gland of experimental rats.

Treatment	C-diet	DWI	BFRC	RCC	BFCC	CCC
*heart*						
Dio1	2.00±0.06^a^	1.96±0.14^a^	2.03±0.12^a^	1.98±0.07^a^	2.15±0.10^b^	1.91±0.07^a^
E2f1	2.21±0.08^c^	2.14±0.15^b,c^	2.07±0.06^a,b^	2.07±0.08^a,b^	2.14±0.06^b,c^	2.01±0.04^a^
Thrα	1.81±0.04^c^	1.67±0.05^a,b^	1.64±0.03^a,b^	1.68±0.05^b^	1.67±0.05^a,b^	1.62±0.02^a^
Thrβ	1.73±0.04^d^	1.66±0.02^b,c^	1.61±0.04^a^	1.64±0.02^a,b,c^	1.67±0.04^c^	1.62±0.03^a,b^
*liver*						
Dio1	1.39±0.02^a^	1.40±0.02^a,b^	1.46±0.02^a,b,c^	1.44±0.02^a,b,c^	1.51±0.01^c^	1.47±0.05^b,c^

Values in rows with different letters (e.g. a, b, c) are significantly different, P≤0.05

C diet—control AIN-93G diet; DWI—diet without iodine in mineral mix; BFRC—diet containing biofortified raw carrot; RCC—diet containing control raw carrot; BFCC—diet containing biofortified cooked carrot; CCC—diet containing control cooked carrot

Dio1 deiodinase iodothyronine type 1

E2f1 E2F transcription factor 1

Thrα thyroid hormone receptor alpha

Thrβ thyroid hormone receptor beta

### Histological analysis

**Fig 1:**
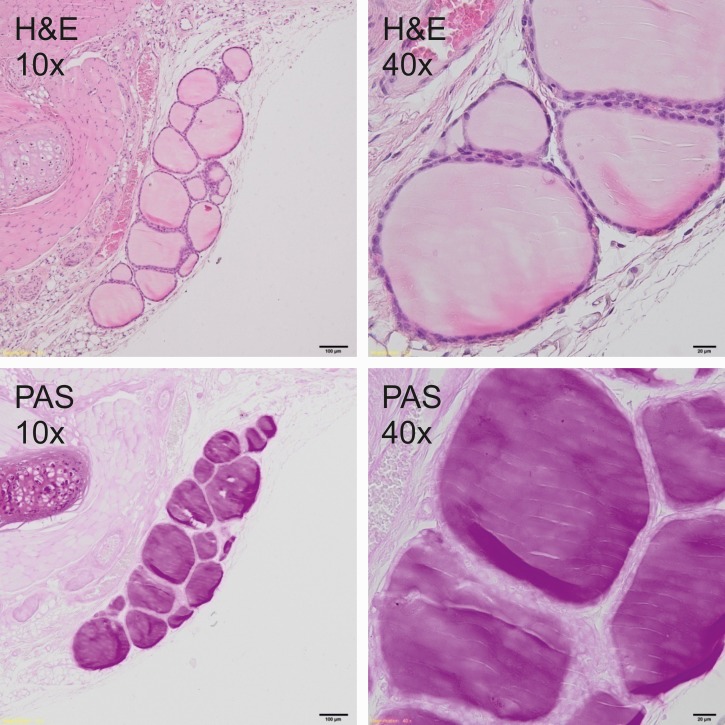
C-group (above–on the left 10x H&S, on the right 40x H&S staining; below on the left 10x PAS, on the right 40x PAS staining).

**Fig 2:**
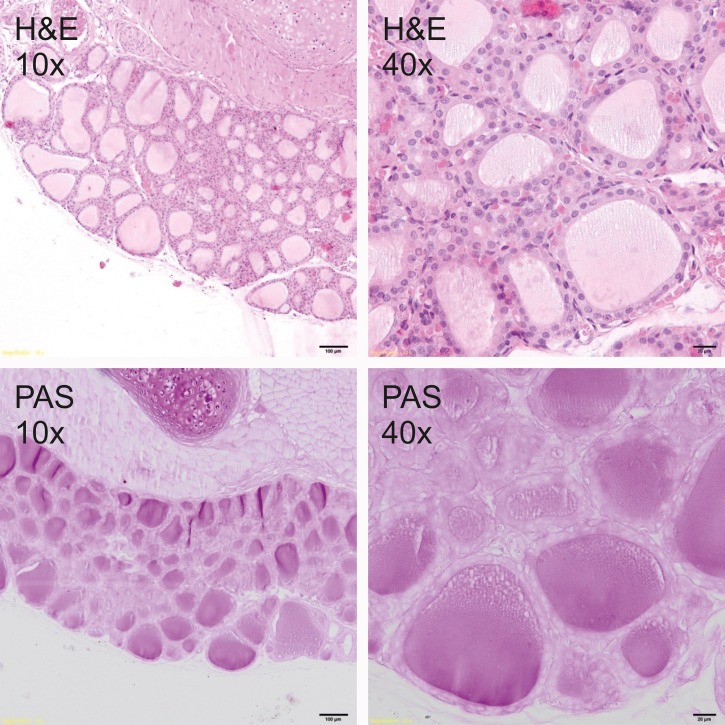
DWI-group (above–on the left 10x H&S, on the right 40x H&S staining; below on the left 10x PAS, on the right 40x PAS staining).

**Fig 3:**
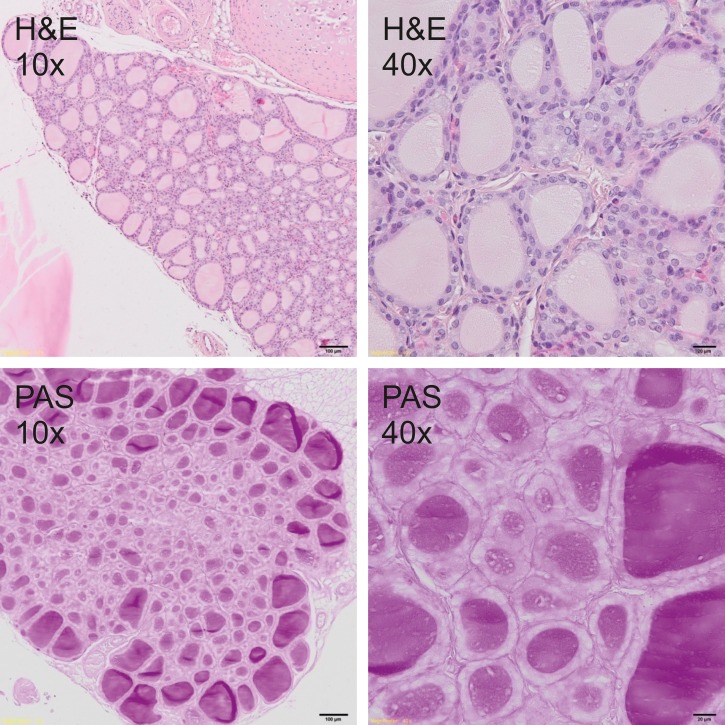
BFRC- group (above–on the left 10x H&S, on the right 40x H&S staining; below on the left 10x PAS, on the right 40x PAS staining).

**Fig 4:**
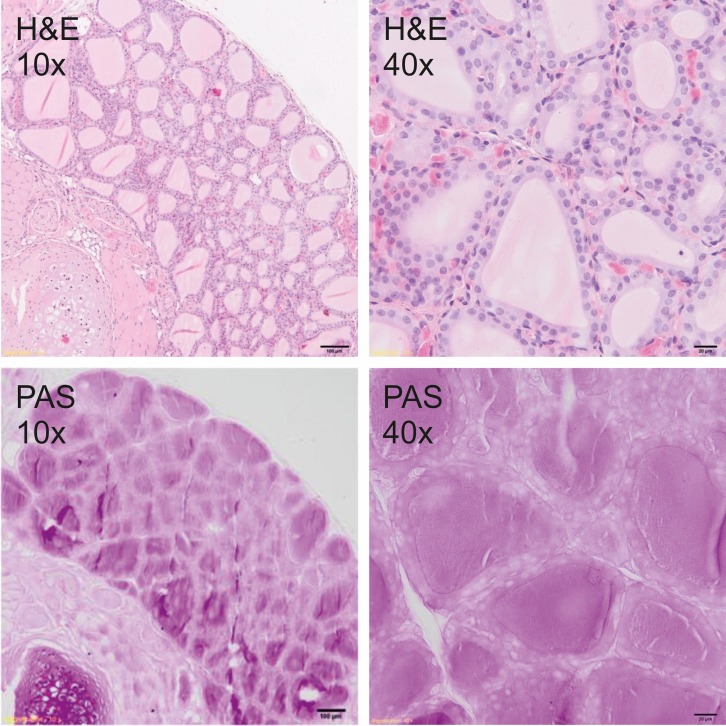
RCC-group (above–on the left 10x H&S, on the right 40x H&S staining; below on the left 10x PAS, on the right 40x PAS staining).

**Fig 5:**
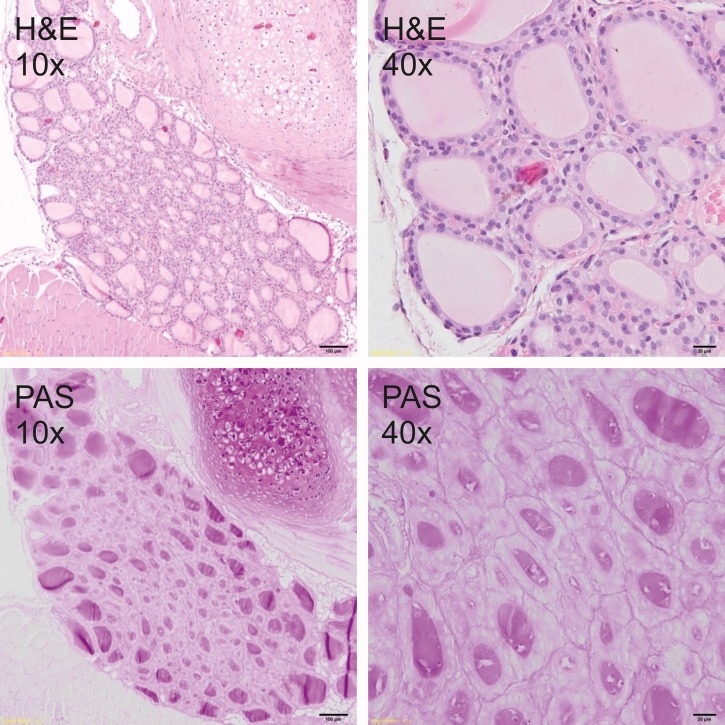
BFCC- group (above–on the left 10x H&S, on the right 40x H&S staining; below on the left 10x PAS, on the right 40x PAS staining).

**Fig 6:**
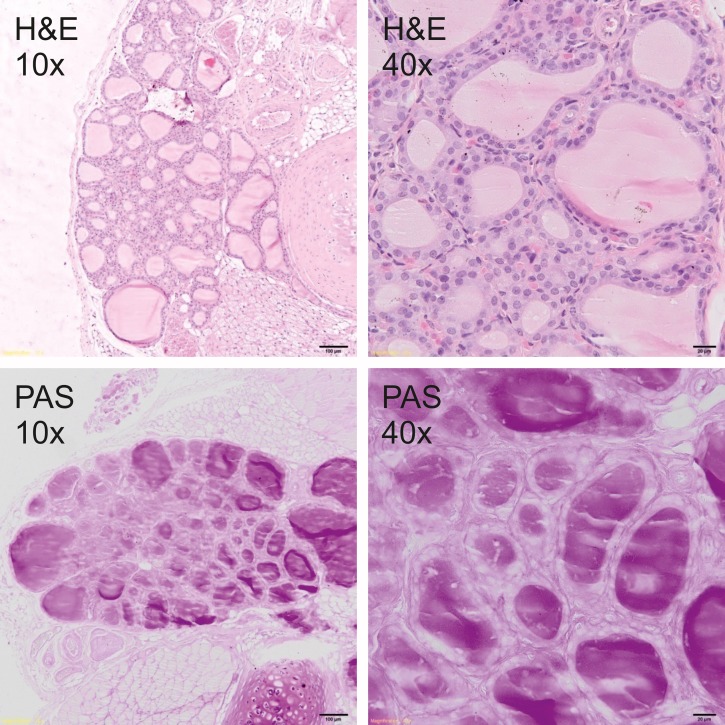
CCC-group (above–on the left 10x H&S, on the right 40x H&S staining; below on the left 10x PAS, on the right 40x PAS staining).

Histological analysis showed a typical inactive thyroid gland in C group demonstrating follicles lined by a single layer of the flattened epithelium and filled with homogenous, eosinophilic (H&E staining, Fig 1) and PAS positive (PAS staining, Fig 1) colloid. In contrast, rodents fed DWI and diets containing carrot as a source of iodine, showed increased secretory activity of thyroid gland, composed of small follicles lined by tall, cuboidal epithelial cells and filled with basophili colloid with single, marginal vacuoles that were observed in DWI group (Fig 2). Among groups fed diets with raw or cooked iodine-biofortified carrots, BFRC group showed strong PAS positive results (Figs 3, 4 and 5), while in RCC group the reaction was weak (Fig 6). There were no differences between BFCC and CCC experimental groups in PAS reaction.

## Discussion

Soil fertilization with iodine and cooking process affected the basic chemical composition of carrots. It is well known that different food processing techniques (fermentation, frying, blanching, cooking) change the amount of nutrients [22–24]. Lower amount of digestible carbohydrates in the control cooked carrots is most likely the effect of partial hydrolysis of starch and leaching of soluble carbohydrates, especially of glucose, during the cooking process. It is difficult to explain why the level of protein and crude fat increased both in the biofortified cooked carrots and in control carrots. Maybe it was caused by changes in dry mass proportions and various nutrients content during cooking process. Probably some water-soluble ingredients (for example glucose, amino-free acids, some minerals including iodine) were extracted during cooking and it caused the increase of the proportion of compounds in dry mass. Albeit it has been reported that in broccoli, cauliflower and carrot the concentration of fat, dietary fiber or indols increased in cooked vegetables in dry mass [25–28]. On the other hand, Yuan et al. [23] reported, based on the fresh mass, some losses of protein, vitamin C and carotenoids in cooked broccoli. The Increased amount of ash is due to biofortification of carrots during the agriculture with KI (potassium iodide). A similar effect reported by Dai et al. [29] showed that iodine concentrations in edible parts of vegetables (pakchoi, spinach, onion, water spinach, celery, and carrot) and the transfer factors of soil-to-edible parts of vegetables significantly increased along with increasing iodine concentration in soil. Hong et al. [30] cultivated four vegetable species (Chinese cabbage, lettuce, tomato and carrot) in soil fortified in iodine (potassium iodide form). They reported that iodine content in each vegetable increased with the increase of iodine levels in soil. Smoleń et al. [31] assessed the influence of soil fertilization with iodine (in the form of iodide I^−^and iodate IO_3_^–^) on the effectiveness of iodine biofortification and mineral composition of carrot storage roots. They obtained the best results in iodine concentration when they used the potassium iodide form of iodine for soil fortification. They also reported that iodine treatment (in both forms: potassium iodide and potassium iodate) had contributed to a significant increase in P, K and Ca content. Our results show that 1000 g of dried, raw biofortified carrots delivered almost 13 mg of iodine and cooked biofortified carrots delivered about 9 mg of iodine. In fresh matter the iodine content is 3.086 mg·1000 g^-1^. Our results show that 100 g of fresh biofortified carrot will deliver 205% of Recommended Nutrient Intake or Recommended Daily Allowance for iodine (about 150 μg I/day for adults) [4]. Therefore, the biofortified carrot could be considered as an excellent source of iodine in daily diets. What is more, vegetables enriched with iodine, especially carrots, which are frequently consumed in many countries around the world, can be an alternative source of iodine for people who should restrict salt intake (salt is the main biofortified product in households of about 70% of the global population) and whose diet is deficient in iodine [32, 33].

The highest level of iodine was observed in raw biofortified carrots. After cooking iodine content decreased. As it was previously mentioned, part of iodine was extracted to water. These results are similar to the data of Comandini et al. [16]. These authors reported losses in iodine during the cooking process of biofortified carrot (25 min. 100°C) amounting to about 56%. The decrease in iodine concentration was also reported by other authors. Rana & Raghuvashi [34] and Longvah et al. [35] reported significant loss of iodine depended on duration of cooking process. To decrease iodine losses during cooking, it is advisable to sprinkle salt on food after cooking rather than to add salt while cooking [34]. Some vegetables with high content of starch may complex some amount of iodine. It is due to presence of V-amylose component of starch in the form of polyiodide chains [36]. Comandini et al. [16] suggested that the low concentration of starch in carrots does not prevent iodine from leaching during boiling, leading to high losses of iodine.

To the best of our knowledge, there is no research concerning the impact of carrots biofortified with iodine on the iodine pathway in an animal study.

The body gain (BG) was affected by different dietary treatments. In RCC and BFCC groups body gain was significantly higher compared to other experimental groups. It can be explained by higher content of carotenoids, especially β-carotene, in RCC diet. Additionally, in those groups FER was significantly higher as compared to rodents fed C-diet and DWI diet. In these diets the addition of carrots was on the level 127.2 g/kg diet. It caused higher concentration of carotenoids in diets. Carotenoids in body are converted to vitamin A, which is necessary for cell division, proliferation and growing of organisms [3]. It could cause higher body gain and FER. In the group fed BFCC diet the highest body gain and FER can be explained by better bioavailability of carotenoids as well as other nutrients from carrots after the cooking process. Kopeć et al. [15] showed that body gain FER, heart and kidney weights were not affected by feeding rats with diets with addition of biofortified or non-biofortified with iodine lettuce. He et al. [37] in a study on pigs fed algae (5 or 8 mg iodine per kg of feed) demonstrated increase (about 10%) in daily body weight gain. On the other hand, Ibrahim et al. [38] reported that hyperlipidemic diet with addition of different tomato products rich in carotenoids did not affect body weight gain and feed efficiency ratio in rats.

Liver weight was the lowest in DWI group as compared to the weight of the liver of rodents fed diets containing carrots, with exception of CCC-diet fed rats. This can be explained by lack of iodine in this diet, and perhaps it limits the adequate development of this organ. Dong et al. [39] reported that diet deficient in iodine affected body size in rat pups. Offspring’s body weight in iodine-deficient group was statistically significantly lower than those of the control group. In our study kidney weight, in contrast to heart and thyroid gland weights, was not affected by various dietary treatments. The control diet caused the greatest increase in weight of these organs. It could be due to variable amounts of both iodine and nutrients and non-nutrients compounds in other diets.

Iodine concentration in urine and faeces was significantly affected by different dietary treatments, which is an important result of our study. It was found that in DWI and CCC groups the excretion of iodine with urine and faeces was the lowest as compared to the other experimental groups, where the level of iodine in diet was adequate. The results of this study suggested that biofortified carrot could be a good source of bioavailable iodine. It was previously reported that concentration of iodine in urine, faeces as well as in selected organs in a rat model can be used for assessment of this trace element content in these animals [40, 41]. A similar effect was observed by Tonacchera et al. [42]. They showed that potatoes, raw carrots, tomatoes, and salad, when bioforified with iodine by foliar fertilization during growing season, provided a significant increase in urinary iodine concentration in humans. It may contribute to the nutritional status of iodine.

On the other hand, we found that the concentration of iodine in urine, faeces as well as in selected organs in the group fed biofortified cooked carrots decreased as compared to groups fed the C-diet and the diet containing raw biofortified carrots (Table 4). This may be explained by the effect of the cooking process which caused not only the loss of iodine during this thermal process, but it could additionally cause changes in iodine bioavailability from biofortified carrots.

It is known that iodine is absorbed in stomach and duodenum and cleared by kidney and thyroid. 70–80% of the iodine body content is located in thyroid gland, the rest in kidney, liver and muscles [43]. Our results showed that the highest iodine concentration in selected organs (femoral muscle, heart, liver, kidney) was observed in rats fed the diet with addition of biofortified raw carrot and the control diet. Additionally, it can be suggested that bioavailability of iodine from raw carrot was better than the one from cooked carrot. Most likely it was due to appropriate iodine content in these diets. It was found that the highest iodine concentration had been detected in kidneys. It may be explained by the metabolic function of this organ. Only few studies discovered changes in iodine concentration in tissues. Kopeć et al. [15] showed that liver and femoral muscle of rats fed iodine biofortified lettuce had the highest level of this trace element as compared to rats fed control diet, as well as a diet containing control non-biofortified lettuce. Hou et al. [44] reported that iodine concentration in skin and hair in healthy adults may be considerably high. Large amounts of this element were also observed in muscles and fat tissue [40].

The level of HDL significantly increased both in groups fed the biofortified raw and cooked carrots and in groups fed the raw and cooked control carrots, as compared to C group (Table 5). It may be explained by the presence of fiber and other biologically active components. However, total cholesterol level was not affected by different diet treatment. We observed an increase in triglyceride level in BFRC rats as compared to rodents fed C- and DWI diets. Carrot is a source of pectin which is a part of soluble dietary fiber [3, 45, 46]. Pectin in colon is fermented by lactic acid bacteria and short chain fatty acids are produced. Short chain fatty acids are absorbed in colon and in liver, which can disturb the metabolism of lipids [47, 48]. Additionally, we discovered that the level of LDL+VLDL has a tendency to decrease in the serum of rats fed BFRC diet. Probably the synthesis of LDL+VLDL that transport TAG from liver, was decreased by the presence of short chain fatty acids and at the same time TAG was removed from liver to the blood, which caused their higher level in the serum of rats. The highest level of TAG in the serum of rats fed BFRC diet can also be explained by higher energy requirements. In this group, the level of T3 was also higher as compared to other experimental groups. It is well known that higher level of thyroid hormones, especially T3, increases the requirement for energy in organisms. It may be suggested that the level of thyroid hormones increased to meet the requirements for energy. It has also been discovered that TBARS level increased significantly in the serum of BFCC diet rats. The cooking process could have increased bioavailability of carotenoids and higher concentration of these compounds in the serum could have caused higher oxidation of lipids and increased level of the TBARS. Additionally, during the cooking process the level of polyphenolic compound probably decreased, which could have also affected higher presence of the products of lipids oxidation. Similar results were presented by Hamza and Mahamoud [49]. These authors reported that adding 15% of fresh carrot to experimental diets decreased TBARS level in liver of male albino rats exposed to gamma irradiation. Different results were presented by Kopeć et al. [15]. They reported that adding lettuce biofortified with iodine and control lettuce significantly decreased TBARs level in the serum of experimental rats.

ALT activity was affected by BFCC and CCC diets. This could have happened due to slight changes in lipid profile in rats from these groups and changes in permeability of the cell membrane, which caused a leakage of this enzyme outside of the cells, leading to higher aminotransferase level in the serum [50].

Adding raw or cooked biofortified carrot to experimental diets did not affect T4 and TSH level in the serum of experimental rats, which is an important finding of our study. It may be suggested that bioavailability of iodine from carrot was sufficient and even if the concentration of this trace element was lower in urine, faeces as well as in selected organs. The organism still had the sufficient amount of iodine to produce thyroid hormones. It was also confirmed by histology (Figs 1–6.). In the thyroid gland of RCC diet fed rats we have found that the reaction with PAS was weak. It can be suggested that the bioavailability of iodine from raw carrots was lower (it was confirmed by lower concentration of iodine in urine, faeces and selected tissues as compared to C-group) and the activity of thyroid gland increased to keep thyroid hormones on the proper level. On the other hand, the level of TSH significantly increased and the level of T3 as well as of T4 significantly decreased in the serum of CCC diet fed rats, which was deficient in iodine. It was also found that the level of T4 significantly increased in the serum of rats fed the BFRC diet and T4 had an increasing tendency (Table 5). It can be suggested that after conversion to vitamin A, carotenoids influenced the production of thyroid gland hormones. There are some research showing that vitamin A level modulates thyroid gland metabolism [51] and that peripheral metabolism of thyroid hormone is necessary for the production of T4 [52] and of thyrotropin or TSH by pituitary [53]. It is known that thyroxin (T4) is a pro-hormone that must be converted to triiodothyronine, which takes place in the liver and kidney. This process is catalyzed by type 1 and 2 deiodinase [54]. Probably, this mechanism is blocked or decreased in CCC group. Additionally, in this group, the iodine excretion was lower. It could be suggested that organism tries to protect the iodine concentration.

In this study we found that mRNA expression of Dio1, E2f1, Thrα, and Thrβ in heart was changed in response to a different diet treatment. In liver tissue we had only found the Dio1 mRNA expression. This expression was also affected by a different dietary treatment. The highest level of mRNA expression was found in BFCC group (both in heart and liver). It is known that Dio1 is mainly present in liver, kidney, thyroid gland, and pituitary. Due to its high activity, the hepatic Dio1 is traditionally regarded as an important source of circulating T3; in turn, its activity is increased by circulating T3 [55]. It could also cause the highest level of T3 in the serum of BFRC group. Our results differ from the results presented by Lavado-Autric et al. [56]. Their data indicate that low-iodine diet induces increases in Dio1 mRNA in thyroids. However, their study lasted 3 months, and perhaps this is the reason why they could observe changes in mRNA expression in this tissue.

Physiological effects of thyroid hormones are principally mediated by hormone action through nuclear receptor proteins that act as ligand-inducible transcription factors and regulate either positively or negatively the expression of target genes in different tissues in a hormone-dependent manner [57]. TRa (thyroid hormone receptor a) is the predominant subtype in bone, gastrointestinal tract, cardiac and skeletal muscle, and central nervous system; TRb (thyroid hormone receptor b) is most abundant in the liver and kidney [58, 59]. In general, TR regulates cardiac rate and contractility [60, 61] while TRb cholesterol regulates homeostasis [62], lipoprotein metabolism [63], and thyroid hormones’ levels [64]. Our results showed that mRNA expression of Thra and Thrb in heart decreased in DWI group and groups with carrot addition as compared to C group. It may be suggested that iodine status and maybe some other bioactive components, especially antioxidants i.e. carotenoids present in carrot, change mRNA expression level.

E2F1 belongs to the E2F family of transcription factor that controls cell cycle by regulating the expression of genes necessary for entering into the S phase [65]; additionally, E2F proteins also regulate gene expression essential for a wide range of other biologic processes, including DNA replication, mitosis, DNA damage repair, differentiation, and autophagy [66, 67]. We observed a decrease in the level of E2f1 mRNA expression in groups fed the diet with either biofortified or control carrots. It may be suggested that carrot addition has affected the cycle of heart cells, probably apoptosis [68].

To conclude, our results demonstrate that biofortified carrot is a good source of bioavailable iodine. Addition of biofortified raw or cooked carrots affects lipid profile, level of thyroid hormones and mRNA expression of selected genes in kidney or liver. Additionally, carrot is a popular vegetable in many countries around the world and can be considered as a potential source of iodine in daily diets for populations with deficiency of this trace element.
